# Dynamics of cilia length in left–right development

**DOI:** 10.1098/rsos.161102

**Published:** 2017-03-08

**Authors:** P. Pintado, P. Sampaio, B. Tavares, T. D. Montenegro-Johnson, D. J. Smith, S. S. Lopes

**Affiliations:** 1 NOVA Medical School Faculdade de Ciências Médicas, Chronic Diseases Research Centre, CEDOC, Universidade Nova de Lisboa, Campo Mártires da Pátria, 130, 1169-056 Lisboa, Portugal; 2 School of Mathematics, University of Birmingham, Birmingham, UK; 3 Institute for Metabolism and Systems Research, University of Birmingham, Birmingham, UK; 4 Centre for Human Reproductive Science, Birmingham Women's NHS Foundation Trust, Birmingham, UK

**Keywords:** cilia length, flow dynamics, left–right development

## Abstract

Reduction in the length of motile cilia in the zebrafish left–right organizer (LRO), also known as Kupffer's vesicle, has a large impact on left–right development. Here we demonstrate through genetic overexpression in zebrafish embryos and mathematical modelling that the impact of increased motile cilia length in embryonic LRO fluid flow is milder than that of short cilia. Through Arl13b overexpression, which increases cilia length without impacting cilia beat frequency, we show that the increase in cilium length is associated with a decrease in beat amplitude, resulting in similar flow strengths for Arl13b overexpression and wild-type (WT) embryos, which were not predicted by current theory. Longer cilia exhibit pronounced helical beat patterns and, consequently, lower beat amplitudes relative to WT, a result of an elastohydrodynamic shape transition. For long helical cilia, fluid dynamics modelling predicts a mild (approx. 12%) reduction in the torque exerted on the fluid relative to the WT, resulting in a proportional reduction in flow generation. This mild reduction is corroborated by experiments, providing a mechanism for the mild impact on organ *situs*.

## 1. Introduction

Arl13b (ADP-ribosylation factor-like protein 13B) belongs to the Ras family of small GTPases and is widely used as a cilia marker [1–3]. The absence of Arl13b induces short cilia, and leads to severe phenotypes in animal models and Joubert syndrome in humans [4–7]. Both in humans and in animal models, the spectrum of phenotypes associated with Arl13b deficiency goes from inappropriate embryonic development to specific changes in the brain, retinal impairment, obesity, incorrect left–right patterning and kidney cysts [4–9]. The characterization of the zebrafish mutant *scorpion*/*arl13b^−/−^* [7] reported very short cilia length and severe left–right pattern defects.

After some years of using overexpression of Arl13b tagged versions in different live embryos [1,3,10], the first study that discussed the impact of Arl13b overexpression in cilia and in embryonic development was published recently [11]. The authors Lu *et al*. [11] showed that *arl13b* expression is induced by Foxj1a [12] and Rfx [13], two major transcription factors involved in ciliogenesis. Arl13b was found to be crucial for cilia length extension, and when its activity was inhibited, even in the presence of these transcription factors, cilia growth was hampered. On the other hand, when Arl13b was overexpressed, cilia length increased and this was sufficient to restore ciliary length in zebrafish embryos deficient in Foxj1 function [11]. These authors reported defective left–right patterning when Arl13b was overexpressed but they did not explore left--right organizer (LRO) fluid flow patterning.

The fluid dynamics of LRO cilia has received significant attention in recent years, including experimental analogues [14], imaging [15–18] and theoretical and computational modelling [18–20]. Similar to the cilia of the mouse node, the tilted rotational motion of cilia is converted to a LR asymmetric flow field [14,21–23] important features in zebrafish Kupffer's vesicle (KV), the fish LRO, include complex ciliation patterns [16] and heterogeneity in cilium length, beat frequency and number [18], which has important implications for the prevalence of normal *situs* [24]. Cilium length in particular has been predicted to be a critical parameter, with models based on a tilted conical rotation pattern predicting that volume flow rate is proportional to the cube of cilium length—therefore, current theory predicts that slightly shorter cilia than observed in wild-type (WT) should produce significantly less flow.

Conversely, longer cilia may be expected to produce a stronger flow than observed in the WT. However, in a study of the target-of-rapamycin (TOR) growth pathway, Yuan *et al*. [25] showed an apparent reduction in flow associated with increased cilium length. TOR complex 1 (Torc1) regulates ribosomal protein S6 kinase 1 (S6k1) activity to influence cilium length; overexpressed S6k1 embryos exhibited cilia with a mildly increased mean length of 7 µm, compared with the WT mean length of a little over 5 µm. Through averaging the path speeds of injected microbeads, Yuan *et al*. reported a dramatic reduction in the counter-clockwise bead trajectories from 11.1 ± 2.3 µm s^−1^ (WT) to 7.0 ± 1.5 µm s^−1^ (S6k1). However, S6k1 overexpression does not only affect cilium length; in addition the beat frequency is reduced approximately 30% from 42.1 ± 3.8 Hz to 29.8 ± 4.9 Hz. Because flow speed is proportional to cilia beat in very low Reynolds number flow, the reduction in beat frequency is likely to be the primary mechanism underlying the reduction in flow velocity in S6k1-overexpressed embryos (42.1/29.8 = 1.41 and 11.1/7.0 = 1.58).

In this study, we used Arl13b overexpression to investigate how increasing cilia length affects fluid flow dynamics and left–right development, while limiting the change in beat frequency to 5%. We focused on the zebrafish KV, a transient organ where both motile and immotile cilia are formed during a developmental period of around 7 h. The KV cells start forming a lumen by two somite stage (11 h post fertilization, hpf) [26]. This lumen is filled with liquid secreted by the surrounding cells involving the CFTR protein [2,27] and will steadily increase in volume until 10–12 somites. In parallel to KV inflation, one cilium per cell gradually protrudes into the KV lumen with around 80% of cilia being motile at 10–12 somites [18].

As a developmental functional assay, we focused on left–right pattern establishment because this developmental step is highly compromised when cilia length is shortened [7,24,28,29] as motile cilia originate inefficient flow that leads to *situs inversus* and heterotaxia. We thus asked if increased cilia length, upon Arl13b overexpression, had any impact on the initial left–right breaking of embryonic symmetry. We observed milder left–right defects than those reported for shorter cilia lengths which prompted us to investigate fluid flow, cilia beat frequency (CBF) and cilia beat pattern for different cilia lengths. This study showed us unpredicted beat patterns of cilia motility that provided a theoretical explanation for the observed data.

## 2. Results

### 2.1. Long cilia mildly influence flow dynamics

**Figure 1.**
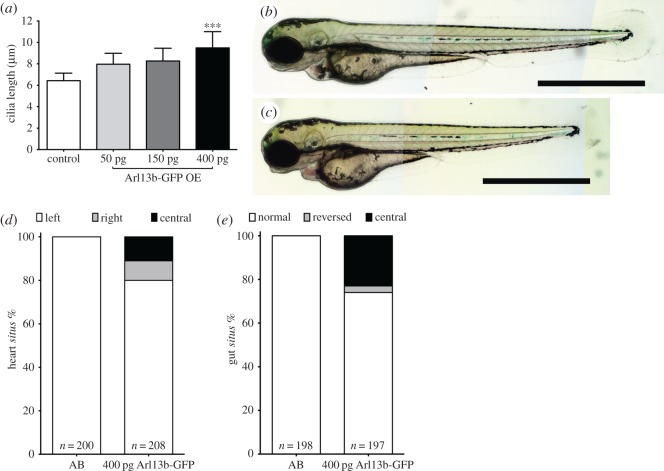
Developmental laterality defects imposed by longer cilia. (*a*) Cilia length increases with growing concentrations of injected messenger RNA for *arl13b*. (*b*,*c*) Wild-type AB zebrafish embryos at 72 hpf, non-injected (*b*), and injected with 400 pg *arl13b-gfp* mRNA (*c*). Scale bar, 1 mm. (*d*,*e*) Laterality of the internal organs: heart *situs* evaluated at 30 hpf (jogging) in live fish (*d*); and gut *situs* evaluated at 53 hpf in fixed embryos by *in situ* hybridization (*e*). Left—left jogging of the heart; right—right jogging of the heart; central—heart tube with no jogging; normal—left liver and right pancreas; reversed—right liver and left pancreas; central—abnormal liver and abnormal pancreas. Source data in http://dx.doi.org/10.5061/dryad.m541q [30].

We titrated the concentration of *arl13b* mRNA injection and found that at 400 pg per embryo there were no signals of toxicity while cilia were already very long (average 9.5 µm s.d. ± 1.5 µm) compared to siblings (6.4 µm ± 1.5 µm) (figure 1*a–c*). We therefore decided to use the 400 pg concentration per embryo for overexpressing Arl13b and characterized the laterality phenotypes and the fluid flow imposed by these long cilia.

Regarding internal organ position, known as organ *situs,* we observed under 20% defects for the heart position and 25% for gut laterality (figure 1*d,e*). These defects are considered mild, taking into account that zebrafish uninjected WT lines can show up to 10% laterality defects. We then measured the flow speed pattern as we described before in Sampaio *et al*. [18] and analysed CBF using high-speed videomicroscopy. Injected embryos showed clearly longer beating cilia with no detectable morphological abnormalities when compared with non-injected embryos (electronic supplementary material, movies 1 and 2, respectively).

Theoretical modelling based on the assumption of straight cilia performing a tilted conical beat suggests that longer cilia should produce substantially more flow. The ‘Q’ formula of Blake and co-workers [23] for flow strength produced per cilium states that

$$Q=\frac{C_{N}(2\pi f)L^{3}\sin^{2}\psi \sin\theta }{6},$$

where *f* is the frequency of the beat in hertz, *C*_N_ is the drag coefficient for normal motion of the rod through the fluid, *ψ* is the half-angle of the cone that the rod traces out and *θ* is the tilt angle. Hence *Q *∝ *fL*^3^: a 1.5 times increase in average cilium length from WT (6.4 µm) to Arl13b-injected (9.5 µm) would lead to a factor of 1.5^3^ = 3.4 increase in the flow strength.

By similar principles, the torque *M* that a straight, rotating cilium exerts on the fluid over the course of a beat is given by

$$M=\frac{C_{N}(2\pi f)L^{3}\sin^{2}\psi }{3}.$$

**Figure 2.**
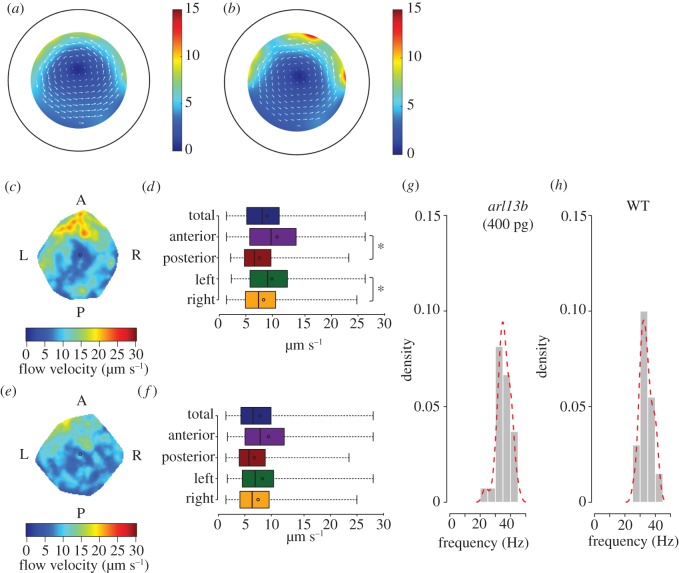
Predicted and observed flow patterns for long cilia. (*a*) Predicted flow map from WT embryos. (*b*) Predicted flow map to *arl13b-*injected embryos. The pseudocolour scale represents flow speed, where red represents high speed versus low speed in blue. (*c*,*d*) Experimental data for WT embryos shown as heat map of flow speed (*c*) and as a box plot for quantification (*d*). (*e*,*f*) Experimental data for *arl13b-gfp* overexpressed embryos (injected with 400 pg of *arl13b-gfp* mRNA per embryo) represented as a heat map of flow speed (*e*) based on the same dataset used to generate the box plot (*f*). The colour scale represents flow speed in µm s^−1^, where red represents high speed versus low speed in blue. Box plots for instantaneous flow speed were based on measurements at different locations of the KV based on 243 tracks for seven WT embryos and 525 tracked native particles in a total of seven embryos overexpressing Arl13b-GFP. (*g*,*h*) Cilia beat frequency average in WT and Arl13b-GFP overexpressing embryos from a total of 30 motile cilia for each condition. Source data in http://dx.doi.org/10.5061/dryad.m541q [30].

This result can be used to model the cilia-driven flow in KV computationally via a distribution of idealized ‘rotlet’ solutions [20]. The resulting flow fields for injected embryos (figure 2*b*) do suggest higher flow speed than WT (figure 2*a*), subject to the assumption that the cilia beat pattern was unchanged. However, our experimental results showed that despite the longer cilia, the flow pattern was still similar to WT, with stronger flow anteriorly and on the left side and was overall 12% weaker than that recorded for WT embryos (figure 2*c*–*f*). Regarding CBF, average values were 33.9 ± 3.48 Hz for WT embryos and 35.7 ± 4.3 Hz for *arl13b-*injected embryos (figure 2*g*,*h*), which were not significantly different (*p* > 0.05).

### 2.2. Cilia-driven flow modelling

Since the predicted increase in flow speed was not observed and CBF was not significantly different, we concluded that the beat pattern of the cilia must be changed in the injected embryos. Since the amplitude of the cilium beat tip *a* = *L* sin *ψ*, the torque-strength formula for a whirling rod can be rearranged as

$$M\propto fLa^{2}.$$

**Figure 3.**
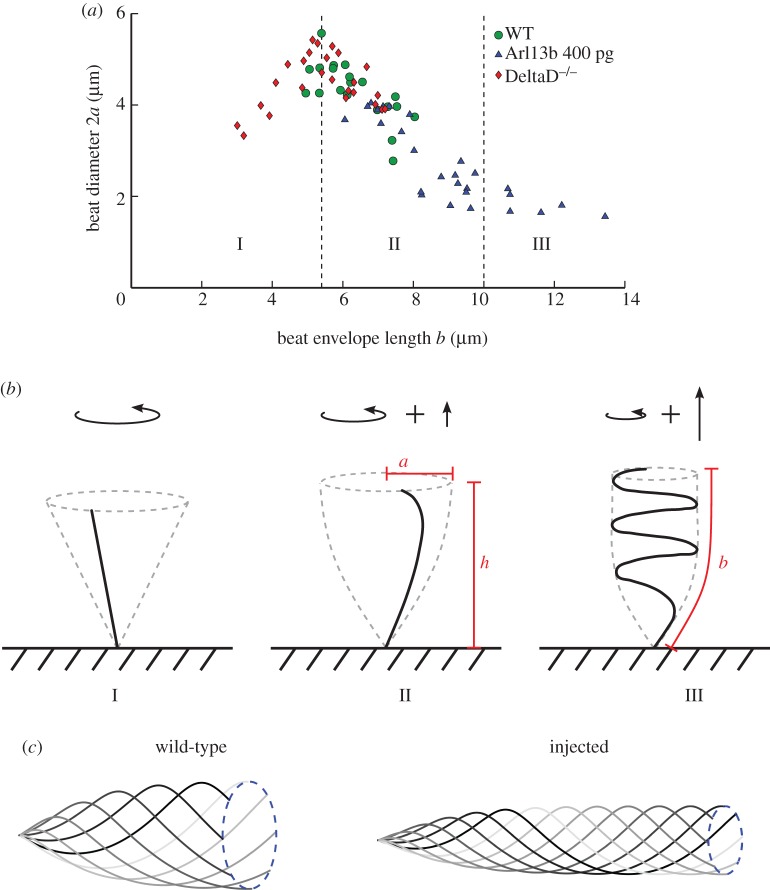
Elastic shape transitions in KV cilia as length increases. (*a*) Cilium beat diameter as a function of the length of the beat envelope as viewed from the side. The plot shows three distinct regions (I) cilia act as straight rods whirling in a conical beat, (II) beat helicity onsets, trailing the cilium tip through the fluid and (III) cilia in fully developed helical beat patterns. (*b*) Short cilia (I), exert a pure torque upon the fluid. As helicity onsets (II), a small force perpendicular to the beat plane is exerted. For fully developed helical beating (III), this force is larger. (*c*) Representative beat patterns used for the fluid mechanics study of WT (left) versus injected (right) embryos (*n* = 30 cilia used for arl13b-injected measurements and for WT). Source data in http://dx.doi.org/10.5061/dryad.m541q [30].

Thus, we see that it is possible to have the same flow strength with longer cilia provided the beat amplitude is decreased. As such, we measured the beat envelope diameter (twice the amplitude) of cilia in WT and *arl13b-*injected embryos, as well as for the short cilia mutant *deltaD^−/−^*, and plotted them as a function of cilium length (figure 3*a*). This plot revealed three distinct regions: (I) beat amplitude initially increases linearly as a function of length, (II) beat amplitude then decreases as a function of envelope length until (III) beat amplitude remains approximately constant as a function of envelope length. Note that in regions (II) and (III), the true length of the cilium is greater than the envelope length, indicating that these cilia are likely curled up in a helical pattern.

These three regions can be understood through the ratio of fluid drag forces to the elastic bending forces in the cilium and the associated effect on cilium beat pattern. As a model of elastohydrodynamic transitions in rotating flagella, Qian *et al.* [31] experimentally and theoretically studied an elastic rod driven to rotate from its base in a viscous fluid. The dynamics can be understood through the dimensionless parameter *χ* = 2*πfηL*^4^/*E* for dynamic viscosity *η* and cilium bending modulus *E*. Qian *et al*. [31] observed a transition from near-straight conical rotation to a helical pattern in the range 5≲χ≲50. We may estimate the mechanical parameters as *E* = 25 × 10^−24^ kg m^3^ s^−2^ [32], *η* = 10^−3^ Pa s, *f* = 34 Hz, and take mean values of *L* = 6.4 µm for WT and *L* = 9.5 µm for *arl13b*-injected. These estimates yields values of *χ* = 14.8 for WT and *χ* = 71.6 for *arl13b*-injected—the order of magnitude for which Qian *et al*. [31] observed the straight-to-helical transition, lending support to this hypothesis.

In region I, cilia are shorter and χ is low; cilia are very rigid and beat out a conical envelope (figure 3*b*). The cilia then reach a critical length of around 5.5 µm and apparently undergo an instability whereby the tip of the flagellum begins to trail behind the main body with a small helical pattern (region II). This instability decreases the beat amplitude at the tip (figure 3*c*), as observed for passive filaments rotated at the base [31] because fluid drag forces on the filament are now higher relative to the elastic forces keeping the filament stiff. Finally, the cilium reaches a length where it folds in on itself into a helical waveform (figure 3*b*, region III). The amplitude of these very long helical cilia does not then change appreciably as a function of length.

The helicity of the cilium beat pattern will have additional effects on the flow field driven by very long cilia, unaccounted for in the simple rotating rigid rod model. For short, rigid cilia (I), the time-average of the cilium beat exerts a far-field straining flow of the fluid [33] with strength proportional to the torque exerted; the effect of the enclosed domain is to convert the straining flow to a global vortex [20]. Longer cilia performing a helical motion exert an additional force along the axis of rotation, as for flagellated bacteria and some spermatozoa, as shown in figure 3. This force is initially small for cilia with small helicity (II), but grows larger as the beat becomes more helical (III). Tilted cilia will exert a component of the force parallel to the cell surface, which has similar long-range behaviour to the straining motion (the component of the force perpendicular to the cell surface will decay rapidly and, therefore, will have a sub-leading influence on the global flow [34]). Thus it is possible that having too many of these, albeit rare, longer cilia might be a factor in the increase in *situs* defects observed in *arl13b-*injected embryos. However, in order to calculate the relative strengths of the symmetry-breaking global vertical flow in WT and injected embryos, we can calculate the torque that an average WT and average *arl13b-*injected cilium exert on the fluid.

Since the average cilium lengths of 6.4 µm and 9.5 µm for WT and *arl13b-*injected embryos, respectively, lie in region II of the cilium beat regime, we expect similar helical beat patterns, with mild helicity for the WT and stronger helicity for the injected cilia. We consider a simple beat parametrization of the form

$$\begin{array}{rl} x & =r(z)\cos\left[2\pi \left(\frac{nz}{h-ft}\right)\right],\\ y & =r(z)\sin\left[2\pi \left(\frac{nz}{h-ft}\right)\right],\\ z & =[0,h]\\ \;\text{and}\;r & =a\left[\frac{1}{1+{\text{e}}^{-(z)}}-\frac{1}{2}\right], \end{array}$$

for time *t*, where *h* is the average height of the untilted cilium tip over the boundary, taking values of *h* = 5.3 µm and *h* = 8.0 µm for WT and injected cilia, respectively, with *a* = 2.2 µm, 1.3 µm, the average radius of the beat amplitude for WT and *arl13b-*injected embryos, respectively. The value of the parameter *n* is then chosen for each of WT and injected embryos so that the total length of the cilia match the average lengths of 6.4 µm and 9.5 µm, respectively. The parameter *n* was found to take the values of *n* = 0.64, *n* = 1.39 for WT and injected embryos, respectively. This produces a simple three-dimensional helical beat from the two-dimensional data that is our best approximation without three-dimensional waveform data of the full beat pattern of a typical WT and injected cilium (figure 3).

Since the vortical flow pattern in the coronal midplane of KV arises from a combination of the torque cilia exert on the fluid [20,21] and cilium tilt, we can use the waveforms given above to calculate the amount of torque that an average WT and an average cilium injected with *arl13b* exerts on the fluid. Using a line distribution of regularized blakelet singularities [35,36], we calculate that for average cilium beat frequencies of 33.9 ± 3.48 Hz (WT) and 35.7 ± 4.3 Hz (injected), in the injected embryos cilia exert just 12% less torque on the fluid than a WT cilium. Since the average strength of flow is proportional to the torque exerted by the cilia, we thus see that the slight decrease in average flow strength of 12%, experimentally measured from WT to injected embryos (figure 3), can be predicted from the change in the waveform kinematics of the cilia.

## 3. Discussion

The fact that longer KV cilia do not have a major effect on flow pattern raises an interesting hypothesis: cilium length may only be a critical factor for lengths below about 5 µm. As we previously showed, the flow dynamics inside the zebrafish LRO is very much affected by short cilia [28] or by less motile cilia [18]. Regarding cilia number, we reason that the crucial difference between mouse and zebrafish is that in the mouse all cilia are ‘useful’ in the sense that cilia are located on the floor of the node, posteriorly tilted and, therefore, all cilia are contributing to a leftward flow (in a time-averaged sense), explaining why two rotating cilia were enough to provide normal left–right markers in mouse as shown by Shinohara *et al.* [37]. The same cannot be said of the zebrafish, as some cilia such as ventral pole cilia are antagonistic to the desired flow (as we explored before in [24] and [20]). In summary, in the zebrafish it is important that flow is produced not only by a certain number of motile cilia, but also by motile cilia that are localized in the productive flow regions of the KV.

Regarding cilia length, this property is a major factor in generating productive flow, the reasoning being that the flow generated by each cilium is proportional to the length of the cilium to the power of 3. By modelling the KV flow pattern with longer cilia based on three-dimensional cilia length measurements, motile cilia number and motility pattern, we could realistically simulate flow dynamics in embryos injected with *arl13b*. The conclusion was that longer cilia adopt a deformed beating pattern that does not result in an appreciably different torque exerted on the fluid in KV when compared with the WT, explaining our observation.

Our results may be compared with those reported by Yuan *et al*. [25] who observed a substantial (approx. one-third) reduction in flow associated with increased cilium length under the conditions of S6k1 overexpression. However, the S6k1 system used by them [25] exhibited reduced CBF simultaneously with increased cilium length. So, from a functional perspective the two treatments are quite different; they [25] manipulated the Torc1 pathway likely to be related to increased intraflagellar transport (IFT) and protein synthesis, while in this study we increase the levels of Arl13b, which is likely to act at the level of the ciliary membrane. Larkins *et al*. [38] performed experiments of fluorescence recovery after photobleaching in IMCD3 cells and showed that dynamics of Arl13b movement within the cilium is not associated with IFT, which is known to cause a fast recovery, but it rather follows a ciliary membrane protein slower dynamics. Therefore, we predict that overexpression of Arl13b-GFP does not affect CBF because it may be independent of the motility machinery present in the axoneme.

In summary, we may say longer cilia do not have a severe effect on LR function; however, there is a limited length of approximately 5–7 µm for which cilia can perform the straight tilted rotational motion, which is optimal for producing LR symmetry breaking. This optimal length seems more tightly regulated towards the short end of the spectrum characteristic of cilia than towards the long end characteristic of flagella, due to simple physical properties of flow and cilia interactions.

## 4. Material and methods

### 4.1. Zebrafish lines

AB WT embryos were raised at 28°C in E3 embryo media and staged accordingly [39]. All procedures with zebrafish were approved by the Portuguese DGAV (Direcção Geral de Alimentação e Veterinária).

### 4.2. Overexpression studies

Arl13b-GFP mRNA microinjections were performed using from 50 to 400 pg per embryo. Human *arl13b* full-length cDNA was fused to GFP and cloned into pCS2+ vector. RNA *in vitro* transcription was done by using the SP6 mMESSAGE mMACHINE Kit (Ambion). Source data are available at http://dx.doi.org/10.5061/dryad.m541q [30].

### 4.3. Heart and gut laterality

At 30 hpf we evaluated heart jogging using a stereoscopic microscope (SMZ745, Nikon Corporation) to observe the embryos from the ventral side. These embryos were then allowed to develop in separated Petri dishes and, at 53 hpf, embryos were fixed and processed for *foxa3 in situ* hybridizations to assess gut laterality as in [28].

### 4.4. Live imaging for flow recording

Mounted embryos between 13 and 14 hpf were set under a 100×/1.30 NA oil immersion objective lens on a Nikon Eclipse Ti-U inverted microscope. The embryos were kept at 25°C overnight in an incubator to delay development and the videos were recorded at room temperature (26°C). All images were taken with the dorsal roof of the KV facing the objective lens. Bright field images were recorded with a FASTCAM MC2camera (Photron Europe, Limited) controlled with PFV (Photron FASTCAM Viewer) software. Native KV particles were filmed at 60 fps for 30 s while cilia were recorded at 500 fps for 2 s. KV flow and CBF measurements were analysed using Fiji software as described previously [18].

## Data accessibility

Data supporting the findings in this paper are available from the Dryad Digital Repository: http://dx.doi.org/10.5061/dryad.m541q [30].
